# Molecular Characterization of Humic and Fulvic Acids of Waterlogged and Well-Drained Amazonian Podzols

**DOI:** 10.1021/acsenvironau.5c00045

**Published:** 2025-09-25

**Authors:** Amanda M. Tadini, Aleksandar I. Goranov, Stéphane Mounier, Débora M.B.P. Milori, Célia R. Montes, Patrick G. Hatcher

**Affiliations:** ■ Embrapa Instrumentação, Brazilian Agricultural Research Corporation − Embrapa, São Carlos, São Paulo 13560-970, Brazil; ‡ Department of Chemistry and Biochemistry, 6042 Old Dominion University, Norfolk, Virginia 23529, United States; § Unité mixte 110, Mediterranean Institute of Oceanography (MIO), Université de Toulon, Toulon 83041, France; ∥ Unité mixte de recherche 7619, Environment, Transfers and Interactions in Soils and Water Bodies (METIS), 27063 Sorbonne Université, Paris 75005, France; ⊥ Unité mixte 110, MIO, Aix Marseille Université, Marseille 13288, France; Unité mixte 110, MIO, Institut de la Recherche et du Développement, Marseille 13288, France; Unité mixte 110, MIO, Institut des Sciences de l’Univers, Centre National de la Recherche Scientifique, Marseille 13288, France; # Instituto de Energia e Ambiente, Universidade de São Paulo, São Paulo, São Paulo 5508-010, Brazil

**Keywords:** Soil organic matter, ultrahigh resolution mass spectrometry, Amazonian soil, podzol, carbon sequestration

## Abstract

The Amazon rainforest is the largest tropical rainforest in the world. Amazonian Podzol soils, characteristic of this region, are known to store substantial amounts of organic carbon in both their surface and deep horizons. Despite decades of research, the molecular-level composition of these soils remains uncharacterized. This study addresses this knowledge gap by employing ultrahigh resolution mass spectrometry, namely, Fourier transform–ion cyclotron resonance–mass spectrometry (FT-ICR-MS), to determine the molecular composition of humic acid (HA) and fulvic acid (FA) fractions from two Amazonian Podzol profiles of varying levels of groundwater exposure (waterlogged vs well-drained). In the waterlogged soil compounds containing nitrogen, sulfur, or phosphorus (NSP) decreased with increasing depth while labile carboxyl-containing aliphatic molecules (CCAM) increased. CCAM were likely preserved through complexation with metals or from kinetically stalled degradation processes. In the well-drained soil compounds containing NSP increased with increasing depth likely due to elevated microbial productivity in the deeper horizons. Oxidation reactions in the well-drained soil profile also led to the production of condensed aromatic compounds (ConAC), which were responsible for the significant carbon sequestration observed in the deeper horizons. The molecular fingerprints of the samples of this study could be successfully parametrized by the nominal oxidation state of carbon (NOSC) derived from FT-ICR-MS suggesting this metric for tracing the podzolization process in future studies of podzol soils. The findings of this study demonstrate the utility of molecular fingerprinting in soil science and emphasize the critical role of hydrology on the molecular composition and carbon dynamics of Amazonian Podzol soils.

## Graphical abstract

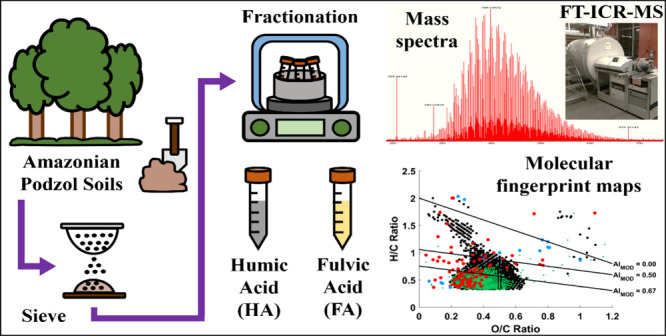

## 1 Introduction

The Amazon rainforest is the largest tropical forest in the world, covering 5–7 million km^2^ and accounting for nearly 40% of the world’s tropical forest area.^1,2^ It is the largest carbon reservoir on Earth, storing 123 ± 23 petagrams of carbon^3^ in its plant biomass and soils.^4–8^ Thus, the Amazon rainforest plays a critical role in the global carbon cycle and is tied to the stability of the global climate. This biome is also one of the most biologically diverse ecosystems on Earth providing critical ecosystem services to humanity.^9–11^

One of the key features of the Amazon rainforest is its diversity of soils.^12^ Amazonian Podzols are of particular interest due to their unique formation process (podzolization) and ability to preserve massive amounts of ancient carbon (10,000 – 50,000 ^14^C years old).^13,14^ These podzols are equatorial hydromorphic soils characterized by thick sandy horizons that overlay clayey horizons. Podzols cover 18% of the Amazon Rainforest^15^ and store 81.4 ± 8.9 Mg ha^–1^ of carbon, which corresponds to 13.6 ± 1.1 petagrams of carbon^14,16,17^ or 11% of the sequestered carbon in the Amazon rainforest biome. However, when disturbed or degraded, Amazonian Podzols may release up to 34 Mg ha^–1^ of carbon from depths of up to 2 m,^16–18^ representing a loss of nearly 42% of their carbon. Such losses contribute significantly to atmospheric carbon emissions and disrupt the global carbon balance. Furthermore, Amazon soils account for 14% of the carbon fixed by the global terrestrial biosphere.^2,19^ This highlights the importance of Podzols in long-term carbon storage and sequestration and necessitates studies on their formation and degradation.

Podzols are formed through podzolization of clayey soils, during which process soil organic matter (SOM) and inorganic ions (mainly of Fe and Al, released through the weathering of various minerals), form SOM-mineral complexes, which are translocated from upper O, A, and E soil horizons down to the deeper B horizons.^20,21^ Therefore, podzol horizons are typically characterized with an eluviated carbon-poor horizon of bleached, ash-gray color located on top of an illuviated dark-colored horizon containing the SOM-mineral precipitates. In previous studies on the Amazonian Podzols of this study we have shown that mobile humic substances, namely the humic acid (HA) and fulvic acid (FA) fractions of SOM,^22,23^ have critical yet contrasting roles in the podzolization process.^13,24,25^ HA fractions in surface A and A-E horizons appear to originate from poorly humified and relatively fresh plant litter residues that were rich in lignin-like compounds. In contrast, HA in the deeper Bh and Bh-C horizons exhibit more variable properties: some soil samples contained recent, but highly humified SOM, while other samples contained much older SOM (>6,000 years) that was poorly humified. These results suggest that the degree of humification of SOM is not strictly related to its age, but rather to its physicochemical characteristics and environmental history.^13^ The FA fractions, due to their much higher solubility and mobility, appear to facilitate the vertical transport of Al, whereas the HA fractions, which are more stable and less soluble, tend to accumulate in Bh horizons and form stable complexes (primarily with Fe). These SOM-Fe complexes contribute to the formation of the dark, carbon-rich spodic Bh horizons typical of podzol soils. The findings of young, poorly humified SOM in deeper horizons reinforce the hypothesis of active dissolved SOM transport throughout the soil profile.^13^ Thus, HA and FA fractions not only reflect, but actively mediate the dynamics of Fe and Al during the podzolization process.

This study aims to further our understanding of the podzolization process by obtaining molecular-level information on HA and FA fractions of well-drained and waterlogged Amazonian Podzols. This is done using ultrahigh resolution mass spectrometry, namely Fourier transform – ion cyclotron resonance – mass spectrometry (FT-ICR-MS), a technique capable of providing detailed molecular fingerprint maps of SOM. In this study, we identify compositional differences between the different soil profiles, their horizons, and their HA and FA fractions. This investigation enhances our knowledge on the role of humic fractions in the genesis of Amazonian Podzols, develops a molecular understanding of their abilities to sequester carbon for millennia, and explores how waterlogging and well-draining affect the podzolization process on the molecular level.

## 2 Materials and Methods

### 2.1 Description of Amazonian Podzol Profiles

Two podzol soils (labeled P1 and P4) were sampled in the upper Rio Negro region of the Brazilian Amazon (north of Barcelos city, Amazon State, Brazil, at coordinates 0°15′33.1″N and 62°46′27.6″W). The soils are classified as Podzol by the standard soil taxonomy system.^26^ This specific geographic region has an equatorial climate, with temperatures averaging about 25 °C, and 3,000 mm of mean annual rainfall. The soils were located in the middle watershed of the Negro River in the Amazon plains of Brazil, nearby the Demini River (Figure 1). The P1 and P4 soils explored in this study have been previously investigated - the exact samples of this study were studied by Tadini et al.^13^ (assessment of humification, recalcitrance, and radiocarbon age), Tadini et al.^24^ (assessment of metals), and Tadini et al.^25^ (assessment of SOM-metal complexation). These soil profiles were also explored by Montes et al.,^14^ who labeled P1 as BAR1 and P4 as BAR4, and explored soil degradation mechanisms such as mineralization and erosion.

**Figure 1:**
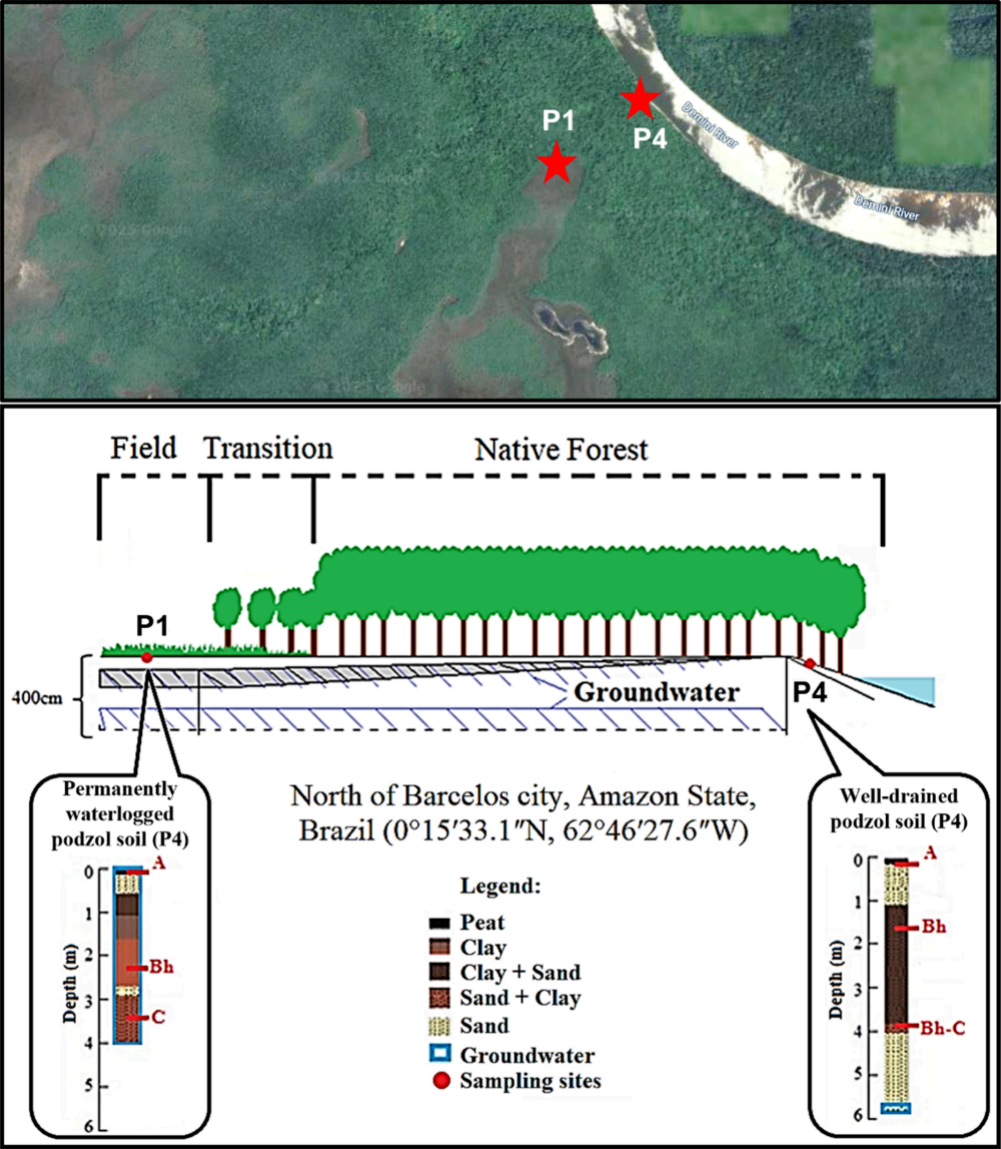
Schematic of soil sampling area. The top panel is an aerial map of the study area (image obtained from Google Maps in July 2025) whereas the bottom panel is an illustration of the sampling area (adapted from Tadini et al.^13^ and Tadini et al.^24^). Adapted with permission from *Science of the Total Environment* [6104210966788]. Copyright [2018] [Elsevier].^13^

The P1 and P4 soil horizons were developed over the quartz-rich sediments of the Rio Negro – Rio Branco Formation (Late Pleistocene), whose unconsolidated sands were due to the formation of an alluvial Megafan associated with the subsidence of the Guiana Shield.^27,28^ Thus, tropical and hydromorphic podzols predominate in the area, with occasional occurrences of ferralsols at isolated elevations.^14,17,20,29,30^ The P1 and P4 profiles exhibit low clay content and minimal aggregation, with bulk densities in surface horizons ranging from 1.20 to 1.53 g cm^–3^.^14^ These two soil profiles represent contrasting hydromorphic environments – P1 is a permanently waterlogged podzol (a hydromorphic podzol) whereas P4 is a well-drained podzol, with no influence of groundwater. Due to their location with respect to the Demini River (Figure 1), the two horizons also have different vegetation cover: P1 is covered by a herbaceous meadow of scrubs and grass (locally known as “campina”) whereas P4 is under rainforest vegetation.

In the waterlogged P1 profile, the reducing conditions limit microbial degradation processes and favor the preservation of poorly humified, lignin-derived compounds. By contrast, the well-drained P4 soil profile shows evidence of significant SOM degradation – SOM in its upper horizons is highly degraded and the lower Bh and C horizons contain older, more recalcitrant SOM that has accumulated over 10,000 years.^13^ This is due to the close proximity of the Demini River, which regularly washes the soil and enhances the mineralization of labile SOM in the upper horizons, but the complete drainage allows for sequestration of stable carbon in the deeper Bh and Bh-C horizons. Furthermore, the meandering waters have completely eroded the upper E horizon making P4 a unique case of a “truncated” podzol soil.^14^ Thus, water-drainage promotes the stabilization of more humified compounds and reduces the abundance of soluble humic fractions, indicating a significant microbial resistance of the accumulating pool of SOM in deeper horizons.

The size distribution of soil particles^31^ further reflects the impact of waterlogging – in P1 the light particles dominate in the surface horizons whereas in the deeper horizons the heavy particles exceed 70% of total SOM indicating a sustained anoxic preservation. Radiocarbon measurements indicated that SOM in the heavy particles of the deep P1 horizons accumulated over 30,000 – 50,000 years indicating a slow carbon accumulation under the sustained waterlogging.^14^ By contrast, heavy particles dominate the surface horizons of P4 suggesting selective mineralization and preferential stabilization of humified structures under the oxic conditions provided by the regular washing by the Demini River.

### 2.2 Soils Sampling and Extraction of Humic (HA) and Fulvic Acid (FA) fractions

Profile P1 was sampled at Horizon A (0–15 cm), Horizon Bh (240 cm) and Horizon C (350 cm). Profile P4 was sampled at horizon A (0–20 cm), Horizon Bh (170–180 cm) and Horizon Bh-C (370–380 cm). Key elemental data for these soils (Al, Fe, and C contents) is shown on Figure S1 of the Supporting Information (SI).

SOM cannot be directly solubilized for analysis by liquid-state techniques, such as FT-ICR-MS. To overcome this limitation, SOM is first base-extracted and subsequently fractionated into FA and HA. These fractions are typically lyophilized into powder form to facilitate further analytical procedures. In this study, HA and FA were extracted from the six soil samples (P1-A, P1-Bh, P1–C, P4-A, P4-Bh, and P4-Bh-C) following the protocol recommended by the International Humic Substances Society.^32^ Briefly, soil was extracted with 0.1 M NaOH at a soil:extractant ratio of 200:1 (mass in g: volume in L). The base-extract was obtained after centrifugation and contained both the HA and FA fractions. The HA was then separated by precipitation at pH 2.0 with 6 M HCl. The FA remained solubilized in the aqueous phase. The FA was purified using a proton-saturated cation exchange resin (Amberlite IR-120, Fluka) and DAX-8 resin (Superlite DAX-8, Supelco). The solid HA was suspended in 0.1 M HCl/0.3 M HF to remove mineral impurities, and then the HA was recovered by centrifugation. This HCl/HF washing procedure was repeated three times. Then, the HA was suspended in water (pH 7) and transferred to dialysis tubes (cellulose membrane dialysis tubing with a cutoff of 14 000 Da, Sigma-Aldrich) to remove any residual halogen ions. The HA suspensions were dialyzed against distilled water until the water tested negative for Cl^–^ with AgNO_3_. At the end, the purified suspensions of HA or solutions of FA were lyophilized to obtain clean powdered HA and FA samples for sequential analytical characterization.

Elemental data and characteristics of HA and FA fractions of these soils from previous studies^13,14,24,25^ can be found in Sections 1 and 2 of the SI (Table S1: elemental composition of HA and FA fractions; Table S2: characteristics of HA and FA fractions).

### 2.4 Negative-Mode Electrospray Ionization–Fourier Transform–Ion Cyclotron Resonance–Mass Spectrometry: (−)­ESI-FT-ICR-MS

#### 2.4.1 Sample Preparation

Briefly, HA samples were solubilized in NaOH at pH = 8 as basic conditions are necessary for HA molecules to solubilize. The solution was then cation-exchanged (CE) to remove Na^+^ and any other cations that were present in the HA. The CE resin was retrieved and was further solvent-extracted with ultrapure methanol to recover any compounds retained onto it as it preferentially absorbs aliphatic compounds.^33,34^ The aqueous fraction (containing most of the HA species) and the methanol extract were combined in a 1:1 ratio allowing all HA species to be introduced into the mass spectrometer. This sample preparation is termed CE_EXTRACTION_ and has been previously found to be most optimal for HA analysis when compared against other methods for HA solubilization prior to negative-mode electrospray ionization—FT-ICR-MS, or (−)­ESI-FT-ICR-MS.^33,34^

FA were prepared by solid-phase extraction (SPE) onto PPL cartridges^35^ and were eluted in methanol. This SPE-PPL method has been also compared by Goranov et al.^33,34^ against other methods for FA solubilization prior to (−)­ESI-FT-ICR-MS and was found to be most effective for the exact same FA samples of this study. More detailed information on the CE_EXTRACTION_ and SPE-PPL preparation techniques can be found in Section 3 of the SI or in our previous publication where they have been described in great detail.^33,34^

#### 2.4.2 (−)­ESI-FT-ICR-MS Analysis

Samples were injected into the Apollo II ESI source of a Bruker Daltonics 10-T Apex Qe FT-ICR-MS instrument at the College of Sciences Major Instrumentation Cluster at Old Dominion University (Norfolk, VA). The instrument is daily calibrated with a polyethylene glycol standard and tune validation was performed using the Suwannee River FA reference material following the recommendations by Hawkes et al.^36^ HA and FA spectra were acquired on two separate days and Suwannee River FA quality control samples were analyzed every 8 h to ensure data comparability (described in Section 4 of the SI). Molecules were ionized in negative ESI mode and 300 scans were coadded to obtain spectra containing >1700 peaks per sample (see Table S3 in SI). ESI voltages were changed on a per-sample basis to obtain spray currents of 13 ± 2 nA ensuring sufficient and consistent molecular ionization. Peak-picking was done using a signal-to-noise ratio threshold of 3.^37^

All acquired spectra were internally calibrated using naturally abundant fatty acids, dicarboxylic acids, and compounds belonging to the CH_2_–homologous series.^38^ The MATLAB-based Toolbox for Environmental Research “TEnvR”^39^ was used for formula assignment and further data analysis (MATLAB version 2022a). Briefly, the *FTMS_RefinementPeaks* code of TEnvR was used to refine the peak lists by removing blank, salt, and isotopologue (^13^C, ^34^S) peaks. Spectra were then aligned to minimize formula assignment errors^40^ using a previously published MATLAB script.^41^ The aligned master spectrum was assigned molecular formulas using the *FTMS_FormulaAssignment* code of TEnvR^39^ using the following elemental criteria: ^12^C_5‑∞_, ^1^H_5–100_, ^16^O_1–50_, ^14^N_0–5_, ^32^S_0–4_, ^31^P_0–2_, with a maximum assignment error of 1 ppm. Formulas that did not adhere to previously established molecular rules for natural organic matter were eliminated.^42–44^ Mass peaks with any residual ambiguous assignments (multiple possible molecular formulas per mass peak) were further refined using inclusion within homologous series (CH_2_, H_2_, COO, CH_2_O, C_2_H_4_O, O, H_2_O, NH_3_) as described by Goranov et al.^39^ Any further ambiguous assignments were refined based on assignment error by selecting the molecular formulas with lower assignment errors. At the end, there was only one molecular formula per mass peak in the final formula catalog of each sample. The acquired formula catalogs of all samples have been published in the *Mendeley Data* repository.^33,34,45,46^

Depending on their elemental composition, molecular formulas are classified in several different compound-like classes^47–53^ based on the presence of N, S, or P nonoxygen heteroelements (i.e., CHO, CHON, CHOS, and CHOP) or based on the biopolymeric class they could represent: Condensed Aromatic Compounds (ConAC), Lignin, Tannin, or Aliphatics.^39^ The number of formulas representing each compound class is presented relative to the total number of assigned formulas.

#### 2.4.3 Statistical Analysis

Principal component analysis was performed by first aligning all molecular formula catalogs using the *FTMS_AlignmentFormulas* code of TEnvR.^39^ Only formulas of 300–800 Da were considered as detection within this mass range is most consistent with the tune configuration of the instrument per the Suwannee River FA quality controls.^36^ Because HA and FA spectral magnitudes were not comparable (due to the different sample preparations), a presence-absence normalization was employed: magnitude values were transformed to the value of 0 (when a formula was not present in a sample) or 1 (when a formula was present). Formula values (1 or 0) were then divided by the number of formulas in each sample to account for differences in the number of formulas detected for each sample.^54^ Using the *Stats_PCA* code, the matrix of aligned and normalized spectra was then used to calculate scores, loadings, and eigenvalues. Additional Pearson correlations were performed using the *Stats_CorrMatrix* code, with a confidence level of 95% used to determine statistical significances.

## 3 Results and Discussion

### 3.1 Molecular Fingerprints of Podzol Humic and Fulvic Acids

In all samples CHO and CHON formulas were the predominant species (41–87% CHO and 8–48% CHON), with the remaining species being CHOS (2–10%) and CHOP (0–7%). The molecular fingerprints were visualized using van Krevelen diagrams^47^ (Figure 2) and revealed several distinct differences between the HA and FA fractions, the two soils (P1 vs P4), and their depth gradients (A through C horizons).

**Figure 2:**
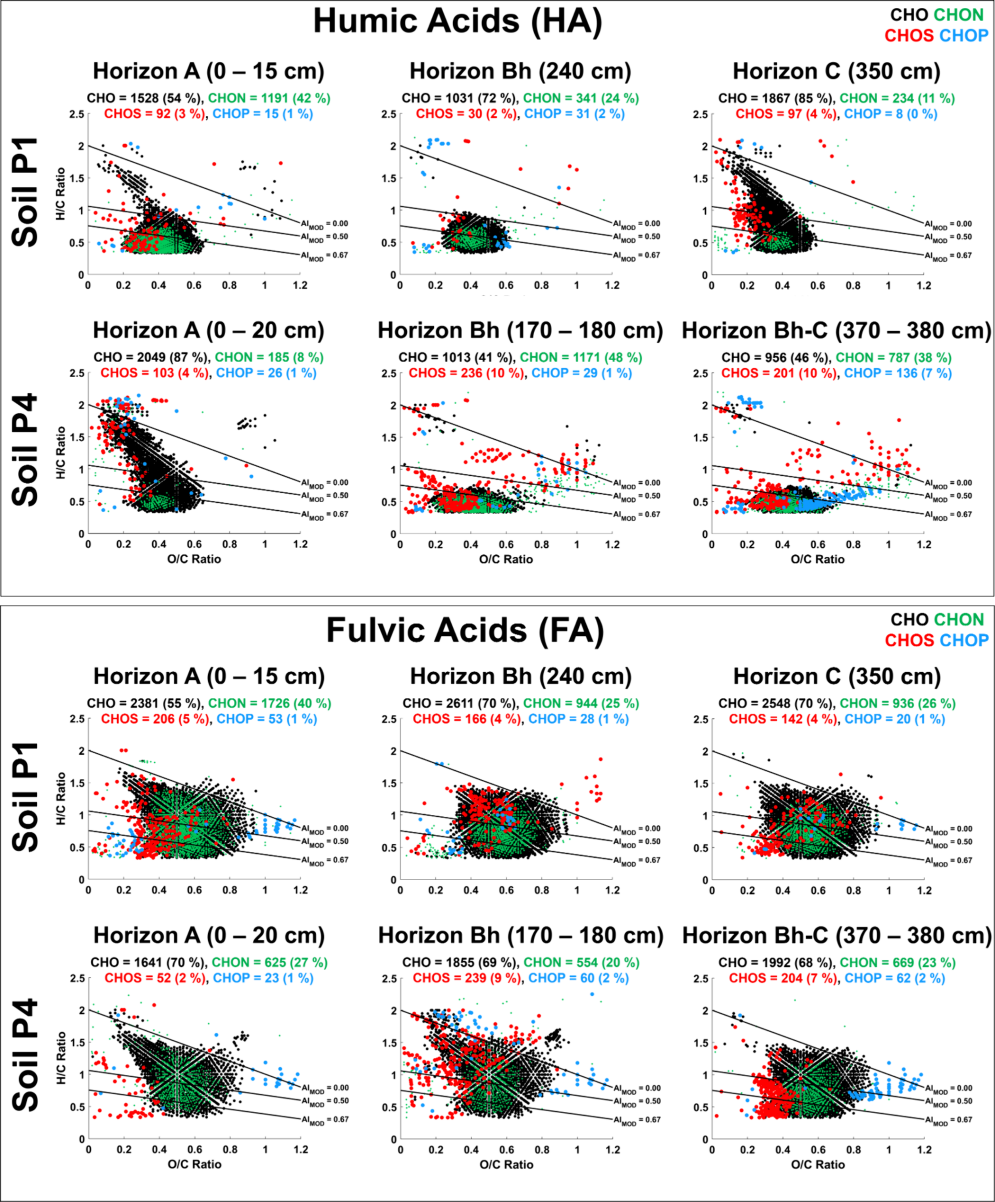
Molecular fingerprints of humic and fulvic acid fractions of soil organic matter of waterlogged (P1) and well-drained (P4) soils. Van Krevelen diagrams (H/C versus O/C plots) show the distribution of CHO, CHON, CHOS and CHOP formula types in samples from different horizons (A through C).

In the waterlogged P1 profile, CHO compounds increased whereas CHON compounds decreased with increasing depth for both the HA and FA fractions. This corroborates well with the elemental analysis (Table S1), which showed decreasing nitrogen content of both the HA and FA fractions in the P1 profile. By contrast, CHO compounds in the well-drained P4 profile generally decreased with increasing depth, while the number of CHON, CHOS, and CHOP compounds increased, also consistent with the elemental analysis (Table S1). This suggests that the hydrologic conditions impact the distribution of N-, S-, and P-containing (NSP) species in podzol soils.

The prolonged interactions between water, SOM, minerals, and microorganisms in the well-drained P4 profile likely stimulate microbial processes, which lead to the generation of more complex, NSP-rich molecules in the deeper horizons. This is supported by previous evidence of high levels of oxygenation, decreasing C/N ratios, and increasing stable nitrogen isotopic composition (δ^15^N) with increasing depth in the P4 soil suggesting strong microbial activities consuming carbon and producing microbially derived SOM.^14^ An alternative interpretation for these observations can be the constant addition of NH_4_^+^ or other N-containing compounds from the Demini River, which can be incorporated into SOM either biotically^55^ or abiotically.^56^ Such biogeochemical processing also explains the observed increases in CHOS and CHOP compounds with increasing depth in the P4 profile.^57^ The waterlogged conditions of P1 appear to minimize these NSP-enrichment processes and lead to the accumulation of CHO formulas in the Bh and C horizons. This could be due to minimal oxygenation (reducing conditions), minimal nutrient availability for microbial growth, NSP-incorporation reactions, root damage and decay (leading to poor carbon cycling), and/or growth of harmful microbes.^58^

Upon further evaluation, the compounds that increase in P1 and decrease in P4 appear to be carboxyl-containing aliphatic molecules (CCAM), which plot in the upper left corner of the van Krevelen diagrams (O/C < 0.4 and H/C > 0.85).^59^ CCAM are lipid-like species hypothesized to be a result of microbial degradation of biomass^60^ but are also a possible byproduct of abiotic oxidation reactions.^59,61^ It has been hypothesized that CCAM are likely intermediates, which could be further humified into more stable forms such as condensed aromatic compounds (ConAC).^59,61^ Due to their high level of oxygenation with carboxyl groups,^62^ CCAM are highly mobile. Therefore, their disappearance in the well-drained P4 soil (Table S4) is likely due to leaching or erosion via the constant washing by the Demini River or potential oxidative conversion to ConAC. Conversely, the waterlogged conditions of P1 likely lead to CCAM preservation, possibly due to kinetically stalled oxidation processes (e.g., poor oxygenation leading to reducing conditions). An alternative explanation could be a possible CCAM complexation with metals, which in tandem with the high Al concentrations (Figure S1) in P1 could explain the preservation of CCAM in this profile and point toward complexation as a stabilization mechanism for CCAM or other forms of carbon in waterlogged soils.

Though often viewed as products of wildfires, highly aromatic compounds (i.e., ConAC) have been known as products of humification for decades, and it has been shown quantitatively that ConAC can be produced nonpyrogenically in massive quantities during naturally abundant oxidation reactions.^63^ Mechanistically, this occurs through Diels–Alder-like cyclopolymerization of muconic acids (lignin oxidation byproducts).^61^ With increasing depth, ConAC decreases in the waterlogged P1 profile but increases in the well-drained P4 profile (Table S4). As the P4 profile is highly oxygenated from the meandering waters of the Demini River,^14^ it has the conditions for the oxidative production of ConAC and its accumulation in the deeper Bh and Bh-C horizons. By contrast, the waterlogged P1 profile is poorly oxygenated and has a noticeable hydrogen sulfide odor indicative of reducing conditions.^14^ Therefore, oxidation reactions must be minimal leading to lower rates of ConAC production and accumulation. These molecular findings and interpretations are well-supported by benzenepoly­(carboxylic acid) marker measurements^64^ providing quantitative evidence for the decreasing ConAC in the deeper P1 horizons and the ConAC accumulation in the deeper P4 horizons (Table S5). Increasing ConAC size in the P4 profile (based on increasing benzenehexacarboxylic acid:benzenepentacarboxylic acid ratios, Table S5) further indicates the presence of oxidation reactions leading to polycondensation reactions forming larger ConAC species.^61^

The molecular findings of this study allow for providing context to previously measured soil basal respiration rates,^14^ which describe the amount of CO_2_ released from soils into the atmosphere through root respiration, biological activity, or oxidation reactions. Basal respiration rates are indicative of the rate of cycling of labile carbon that rapidly renews in surface soil layers.^65^ There is a higher proportion of labile carbon in the waterlogged P1 soil than in the well-drained P4 soil indicative that waterlogging preserves labile carbon and shields it from mineralization.^14^ The observation of stabilized CCAM in the waterlogged soil suggests that some or all of this labile carbon is CCAM, which reveals the potential biogeochemical reactivity of these types of molecules in SOM. Future studies employing biological soil incubations should empirically test this and determine the ability to CCAM to degrade to CO_2_, transform to ConAC,^61^ and sequester SOM in waterlogged conditions.

### 3.2 Podzolization and Its Relationship with Molecular Trends

While evaluating the molecular formula distributions of individual samples is informative (e.g., via van Krevelen diagrams, Figure 2), it is difficult to determine the most important molecular traits throughout the podzolization process with increasing depth. Principal component analysis is an excellent tool to deconvolute multivariate data sets like ours and find the most important trends.^66^

The scores plot (Figure 3A) revealed that HA and FA are of vastly different composition as their scores separated along PC1, which explains 28% of the total variance. This result aligns well with our expectations, as HA and FA are operationally defined SOM fractions known to differ substantially in molecular composition as consistently reported in the literature for decades, including in previous studies on the same soil types and samples.^13,14,24,67^ Additionally, the differing chemical matrices of HA and FA necessitated distinct sample preparation protocols prior to (−)­ESI-FT-ICR-MS analysis (see Section 3 of the SI). Different sample preparations lead to different biases in the detected ion species^33,34^ contributing to some extent to the pronounced separation observed along PC1 (*x*-axis of Figure 3A).

**Figure 3:**
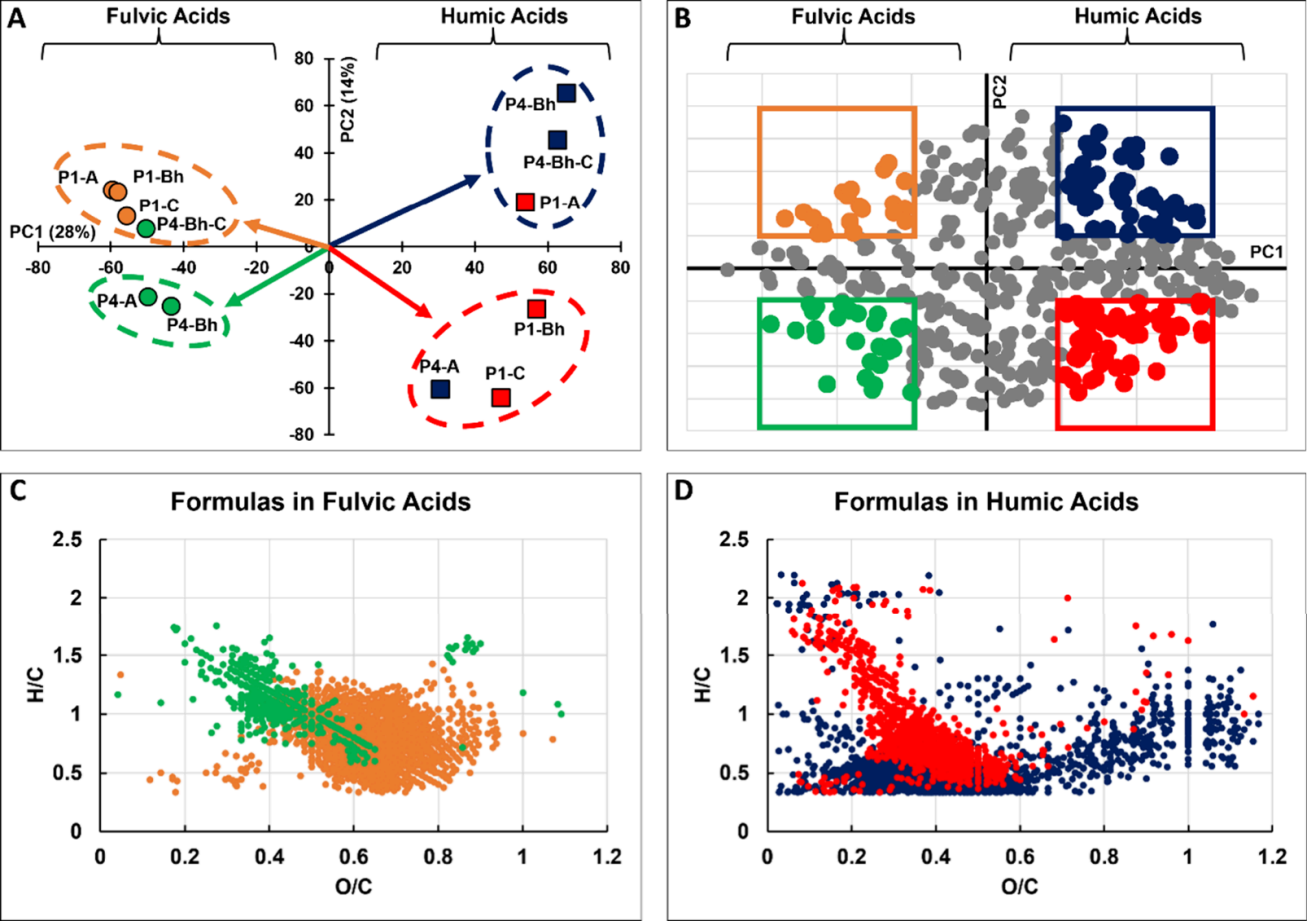
Principal component analysis of molecular data for Amazonian podzol humic and fulvic acids. Panel A shows scores corresponding to each sample for the first and second principal components (PC1 and PC2, respectively, with the explained variance by each component shown as%); panel B shows the loadings (gridlines every 0.005 units) with the significant loadings being colored whereas insignificant loadings being in gray (significance determined by colocation instead of p-values, see Figure S4). Panels C and D show van Krevelen diagrams of the molecular formulas corresponding to the color-coded loadings from panel B. Additional plots can be found in section 6 of the SI: Figure S4 showing data exploration using p-value significance determination; Figure S5 showing that PC2 and the third principal component (PC3) are correlated (i.e., no new information can be obtained from exploring PC3); and Figure S6 showing the molecular weight distributions of the formulas displayed on panels C and D.

FA samples (Figure 3C) showed the presence of lignin-like molecules whereas HA samples showed the presence of much more aromatic compounds (at lower H/C values; Figure 3D) in agreement with the general postulate that HA fractions are more aromatic than FA fractions. The FA of the two soil profiles noticeably differed in degree of oxygenation (i.e., O/C) – FA species of the waterlogged P1 soil were more oxygenated (i.e., having formulas at higher O/C values) whereas FA species of the well-drained P4 soil were less oxygenated (i.e., having formulas at lower O/C values). This is likely due to the regular washing of the P4 soil leading to the mobilization of oxygenated compounds into the Demini River leading to the apparent enrichment in poorly oxygenated compounds. FA formulas in the A and Bh horizons of P4 were also of smaller molecular weight (<650 Da; Figure S6) indicating they are likely freshly produced^68–70^ further agreeing with their proposed microbial sourcing.^14^ By contrast, FA formulas in P1 were of various molecular weights (300 – 800 Da, Figure S6) indicating that waterlogging can preserve SOM of various sizes. Noticeably, the FA of the deepest P4 horizon (Bh-C) clustered with the FA samples of P1 indicating that the leaching and biochemical degradation of SOM in the P4 profile become stalled before the Bh-C horizon, e.g., at around 3 m (Figure 1).

HA samples contained distributions of ConAC, some highly aromatic lignin-like compounds (likely polyphenols), oxidized lignin-like or tannin-like compounds, as well as CCAM (Figure 3D). N-containing lipid-type formulas were also observed, which likely correspond to nitrogenous lipids such as glycosphingolipids.^51,53^ HA of the deeper P1 horizons Bh and C, as well as the HA from the surface A horizon of P4 separated along the PC2 axis due to their higher content of CCAM and polyphenols. These compounds, the polyphenols in particular, are generally viewed as fresh residues of plant litter that have not yet experienced humification. This further suggests that waterlogging preserves fresher compounds in the deeper P1 horizons. Such compounds also exist in the surface P4 horizon, but do not sequester in the deeper horizons. The remaining HA samples (of the surface A horizon of P1 and of the deeper Bh and Bh-C horizons of P4) separated on the other end of the PC2 axis (Figure 3A) indicative of a higher content of ConAC, highly aromatic polyphenols, and tannins (Figure 3D). These horizons do not experience waterlogging (from groundwater or the Demini River) leading to production and sequestration of stable molecules such as ConAC.

To quantitatively assess why the HA and FA samples of this study differed and separated distinctively along the principal coordinates, score values (Figure 3A) were investigated with respect to sample chemical properties. As the separation across PC1 was primarily caused by the intrinsic differences in extraction and sample preparation, PC1 scores were not evaluated as they would not provide meaningful information for the differences among soils, horizon depths, or hydrologic conditions. The second principal component (PC2) scores, however, do appear to provide meaningful biogeochemical information and thus, were correlated with H/C, O/C, C/N ratios from elemental analysis or key metrics derived from molecular formulas (Figure 4). The H/C ratio can serve as a proxy for aromaticity,^47,53,71^ the O/C ratio can serve as a proxy for oxidation,^59,61,72,73^ and the C/N ratio can serve as a proxy for biodegradation.^74–76^ Mass spectrometry indices include molecular weight (MW), the aromaticity metrics double-bond equivalency (DBE) and modified aromaticity index (AI_MOD_),^77^ nominal oxidation state of carbon (NOSC),^78^ and labile formulas above the molecular lability boundary (%MLB_L_).^79^ All of these molecular metrics are sensitive to different biogeochemical processes and can be used to investigate what causes SOM to change across the two different soil profiles of this study.

**Figure 4:**
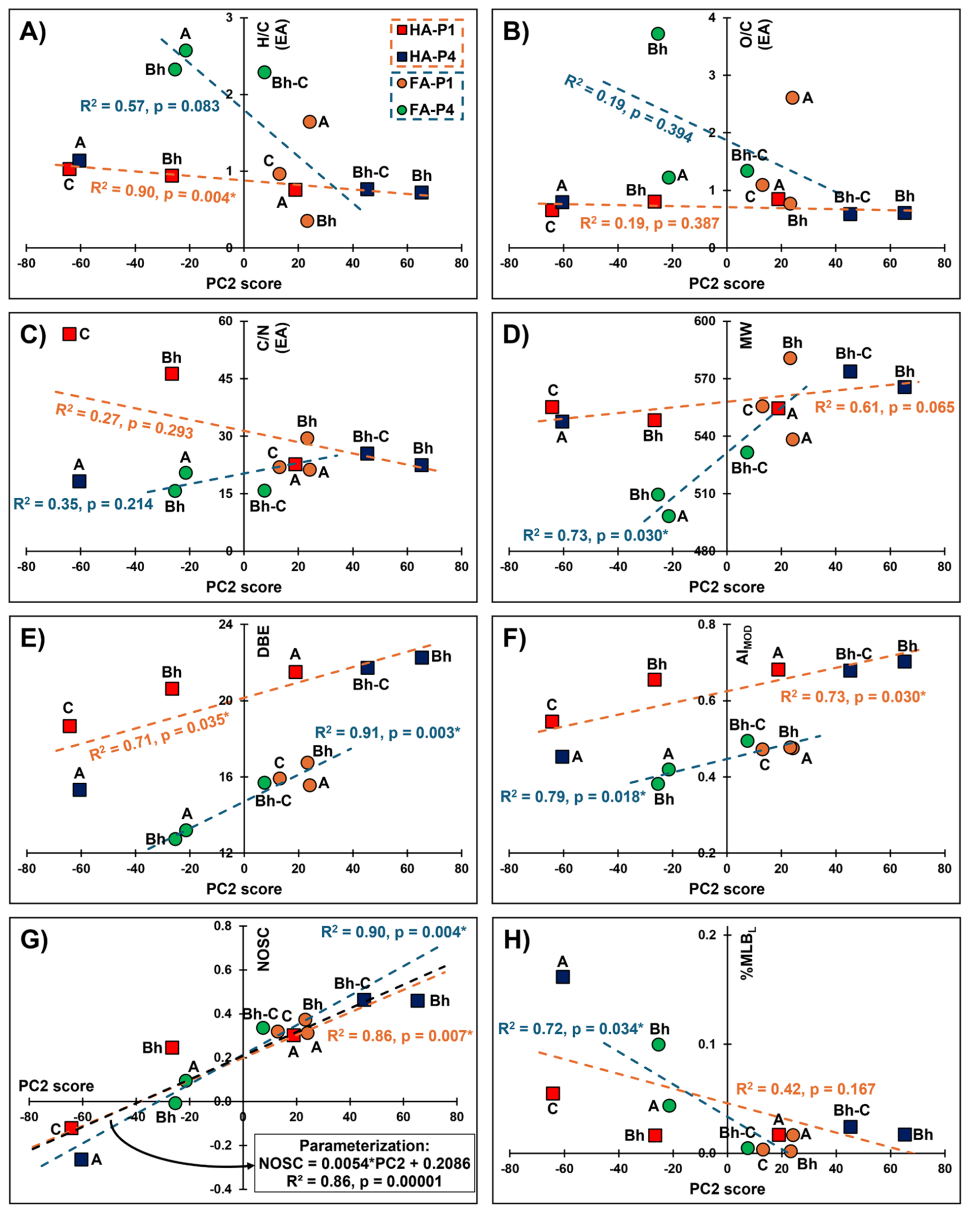
Assessment of second principal component (PC2) scores relative to different sample parameters derived either from elemental analysis (panels A = H/C, B = O/C, and C = C/N ratios) or ultrahigh resolution mass spectrometry (panels D = molecular weight, E = double-bond equivalency, F = modified aromaticity index, G = nominal oxidation state of carbon, and H = percentage of formulas above the molecular lability boundary). Samples are color-coded based on type of sample (humic vs fulvic acid) and soil profile (waterlogged P1 vs well-drained P4). Humic acid (orange trendlines) or fulvic acid data (blue trendlines) were correlated using Pearson correlations, with significant p-values (*p* < 0.05) labeled with an asterisk (*). Given that the humic and fulvic correlations overlapped when their NOSC values were explored, a global correlation of all data was developed (black trendline in panel G).

The investigation of molecular composition against sample properties (Figure 4) revealed that the PC2 scores are best explained by differences in degree of SOM oxidation (parametrized by NOSC, Figure 4G). Similar correlations were obtained when assessing the third principal component (PC3), because it correlates well (*R* ^2^ > 0.87) with PC2 (Figure S5). While many sample metrics had significant Pearson correlations with the PC2 scores (*p* < 0.05), only NOSC showed similar correlations in both HA and FA samples. This allowed to develop a “global” correlation to judge SOM properties regardless of HA/FA fractionation or different sample preparation technique prior to (−)­ESI-FT-ICR-MS analysis. Therefore, NOSC could be investigated as a metric for tracing humification and podzolization processes in podzol soil profiles. While NOSC here is derived from (−)­ESI-FT-ICR-MS, similar carbon oxidation metrics can be derived from elemental analysis or solid-state nuclear magnetic resonance spectroscopy,^80^ techniques that are much more widely available.

These results indicate that oxidation, as parametrized by NOSC, is likely the most important mechanism during the podzolization process. Previous studies have also pointed toward oxidation as a key mechanism during soil podzolization. Using infrared spectroscopy, Do Nascimento et al.^81^ identified the presence of COOH and OH groups linked to clay minerals in Bh horizons suggesting that oxygen-rich compounds (e.g., such as CCAM) bind with Fe and Al leading to their removal. Using pyrolysis coupled with gas chromatography–mass spectrometry, Brock et al.^82^ observed a decrease in phenolic compounds and increase in aliphatic compounds along a podzolization gradient. This is suggestive of oxidative degradation as upon oxidation phenols and ConAC transform into aliphatic compounds.^83^ Another study from the Amazon Basin demonstrates that as SOM accumulates in Bh horizons during Podzol development, SOM is oxidized and mobilized into deeper horizons, in parallel with sourcing dissolved organic matter to nearby fluvial systems.^84^ Collectively, our molecular findings contextualized with existing literature point strongly toward oxidation being a key chemical pathway during the podzolization process. This provides a mechanistic explanation of the different stages of podzolization (hydromorphic regulation, organo-metallic transport, long-term SOM transformation) allowing us to better understand regional biogeochemistry in podzol systems and their carbon dynamics. However, it must be noted that NOSC could simply be changed by a selective removal of compounds, which could be the case during SOM leaching in the P4 profile and likely explains why NOSC trends are not always correlated with increasing depth (Figure 4G). Therefore, future studies should design causal laboratory experiments to test the exact mechanistic pathways within the podzolization process and empirically determine the role of oxidation in the podzolization mechanism.

### 3.3 Environmental Significance

Soils exposed to different hydrologic conditions play a crucial role in both sourcing dissolved organic matter to fluvial environments^85^ and in stabilizing SOM in deeper horizons. During humification, plant biomass from topsoil horizons is reworked by microorganisms and oxidative processes, which yields larger, more recalcitrant molecules that are stabilized in the deeper soil layers.^59,86^ This explains the high degree of SOM aromaticity observed in deeper podzol horizons (Figure 2). Our investigation shows that varying geomorphological characteristics (e.g., waterlogging, flooding) influenced the biogeochemical processing of SOM leading to differences in molecular composition and accumulation of SOM in podzol soils (Figures 2 and 3). The obtained SOM molecular fingerprints provided chemical context to the preservation of labile carbon in the waterlogged P1 soil and the accumulation of refractory carbon in the well-drained P4 soil.^13,14^

Molecular fingerprinting with (−)­ESI-FT-ICR-MS allowed to suggest the likely SOM types of compounds participating in the different mechanisms within the podzolization process. Figure 5 is a conceptual diagram describing the humification process in the podzol soils of this study summarizing our molecular findings. As plant litter is processed, the labile biopolymers (e.g., carbohydrates, proteins) are mineralized leaving small aromatics, such as lignin and tannins, to be abundant in surface soil horizons (e.g., O, A, E). Upon further humification and translocation of SOM down the profile through podzolization, larger molecular classes, such as CCAM and ConAC, are formed. It appeared that in well-drained soils (e.g., P4) carbon is actively cycled and transformed into ConAC. Thus, this group of compounds is likely responsible for carbon sequestration. By contrast, in waterlogged soils (e.g., P1) the kinetics appear stalled, which leads to the preservation of more labile material, such as CCAM, which appear to be intermediates prior to their conversion to ConAC. These differences in biogeochemical processing significantly impact long-term soil and water biogeochemical properties exemplifying the importance of molecular investigations in soil science.

**Figure 5:**
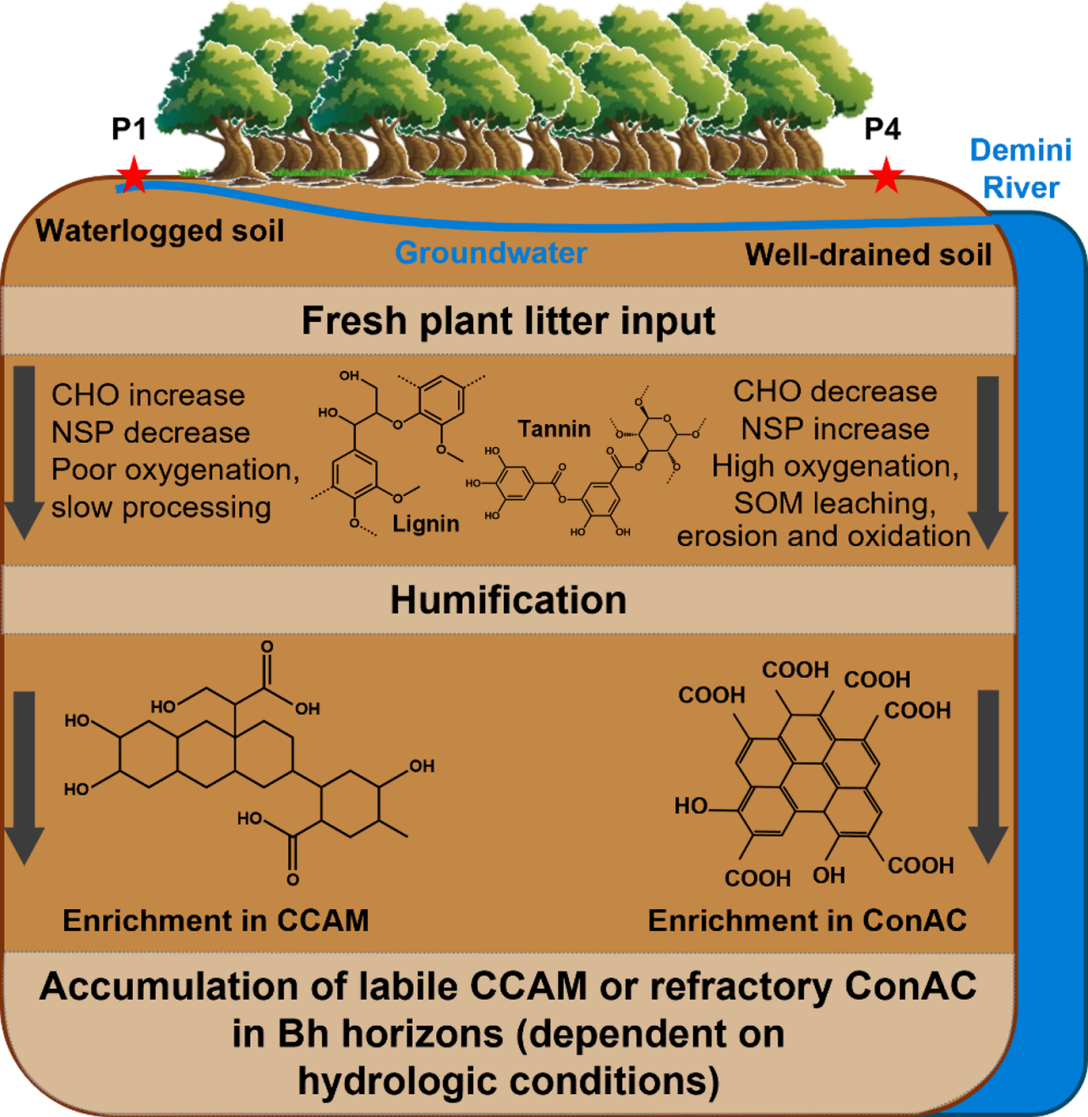
Conceptual diagram illustrating the transformation and stabilization of soil organic carbon with increasing depth in Amazonian Podzols. For simplicity, only classes of importance to the podzolization process are shown (lignin, tannins, CCAM = carboxyl-containing aliphatic molecules, ConAC = condensed aromatic molecules).

## 4 Conclusion

In this study, electrospray ionization–Fourier transform–ion cyclotron resonance–mass spectrometry (ESI-FT-ICR-MS) was employed to characterize soil organic matter of two Amazonian podzol soils of different hydrologic conditions (waterlogged vs well-drained). The comparison of molecular fingerprints of humic and fulvic fractions of different soil horizons demonstrated that the hydrological conditions directly influence the nature of organic compounds sequestered in deeper profiles of Amazonian Podzols. The well-drained P4 profile exhibited a higher abundance of condensed aromatic, nitrogen-, sulfur-, and phosphorus-rich compounds. These appear to be formed through oxidation and/or microbial degradation pathways leading to significant accumulation of degradation-resistant carbon in the form of condensed aromatics that become translocated through podzolization and sequestered in deeper horizons. By contrast, the waterlogged P1 profile was characterized by a greater presence of carboxyl-containing aliphatic molecules, which appeared to have been stabilized due to kinetically stalled degradation processes or by complexation with metals. Principal component analysis supported these findings and indicated that the nominal oxidation state of carbon could potentially be a useful parameter for tracking humification and podzolization processes in podzol soils. These results demonstrate the utility of ESI-FT-ICR-MS and emphasize on the role of carboxyl-containing aliphatics and various aromatic compounds in Amazonian Podzols and their role in stabilizing carbon in deeper soil horizons under different hydrologic conditions.

## Supplementary Material



## Notes

Elemental composition data of soil and humic fractions; description of the importance of humic substances in podzolization processes; additional details on sample preparation and mass spectrometric analysis of humic fractions; benzenepoly­(carboxylic acid) measurements of condensed aromatic carbon; and supplemental figures on the statistical analysis (PDF)
