# Supplementary material for: Molecular Characterization of Humic and Fulvic Acids of Waterlogged and Well-Drained Amazonian Podzols
Source: ACS Environ Au. 2025 Sep 25;5(6):561–72. doi: 10.1021/acsenvironau.5c00045 (PMC12635939; doi:10.1021/acsenvironau.5c00045)
Supplement: Supplementary file 1 [file vg5c00045_si_001.pdf]

Supporting Information (SI)

Molecular characterization of humic and fulvic acids of waterlogged and well-drained Amazonian Podzols

Amanda M. Tadini[a,b,c,d*#], Aleksandar I. Goranov[b#], Stéphane Mounier[c,e], Débora M.B.P. Milori[a], Célia R. Montes[f], Patrick G. Hatcher[b]

[a]Embrapa Instrumentação, Brazilian Agricultural Research Corporation – Embrapa, São Carlos, São Paulo 13560-970, Brazil

[b]Department of Chemistry and Biochemistry, Old Dominion University, Norfolk, Virginia 23529, United States

[c]Unité mixte 110, Mediterranean Institute of Oceanography (MIO), Université de Toulon, Toulon 83041, France

[d]Unité mixte de recherche 7619, Environment, Transfers and Interactions in Soils and Water Bodies (METIS), Sorbonne Université, Paris 75005, France

[e]Unité mixte 110, MIO, Aix Marseille Université, Marseille 13288, France; Unité mixte 110, MIO, Institut de la Recherche et du Développement, Marseille 13288, France; Unité mixte 110, MIO, Institut des Sciences de l'Univers, Centre National de la Recherche Scientifique, Marseille 13288, France

[f]Instituto de Energia e Ambiente, Universidade de São Paulo, São Paulo, São Paulo 5508-010, Brazil

*Corresponding author e-mail: amandatadini@alumni.usp.br

[#]These authors contributed equally to this work (A.M.T., A.I.G.)

Section 1. Elemental composition data of soil and humic fractions:

Figure S1 shows aluminum (Al) and iron (Fe) concentrations across different soil horizons of the two Amazonian Podzol profiles (P1 and P4), along with carbon content (% C). The highest Al concentration is observed in the Bh horizon of profile P1 (92 g/kg), with all other samples having significantly less Al, ranging 41 – 52 g/kg. The Bh horizon of P1 also had the highest Fe content (6.4 g/kg), with all other samples having significantly less Fe, ranging 1.2 – 2.5 g/kg. With regards to the organic matter content, the A horizon had the highest carbon content (22.5 %), which decreased down to 1.2 % in the deeper horizons. The P4 profile had considerably less organic matter, with carbon content being 3.9 % in the surface (A) horizon and depleting down to 0.38 – 0.48% in the Bh and Bh-C horizons due to the significant degradation that this profile experiences. Further details are available in Tadini et al.[1]

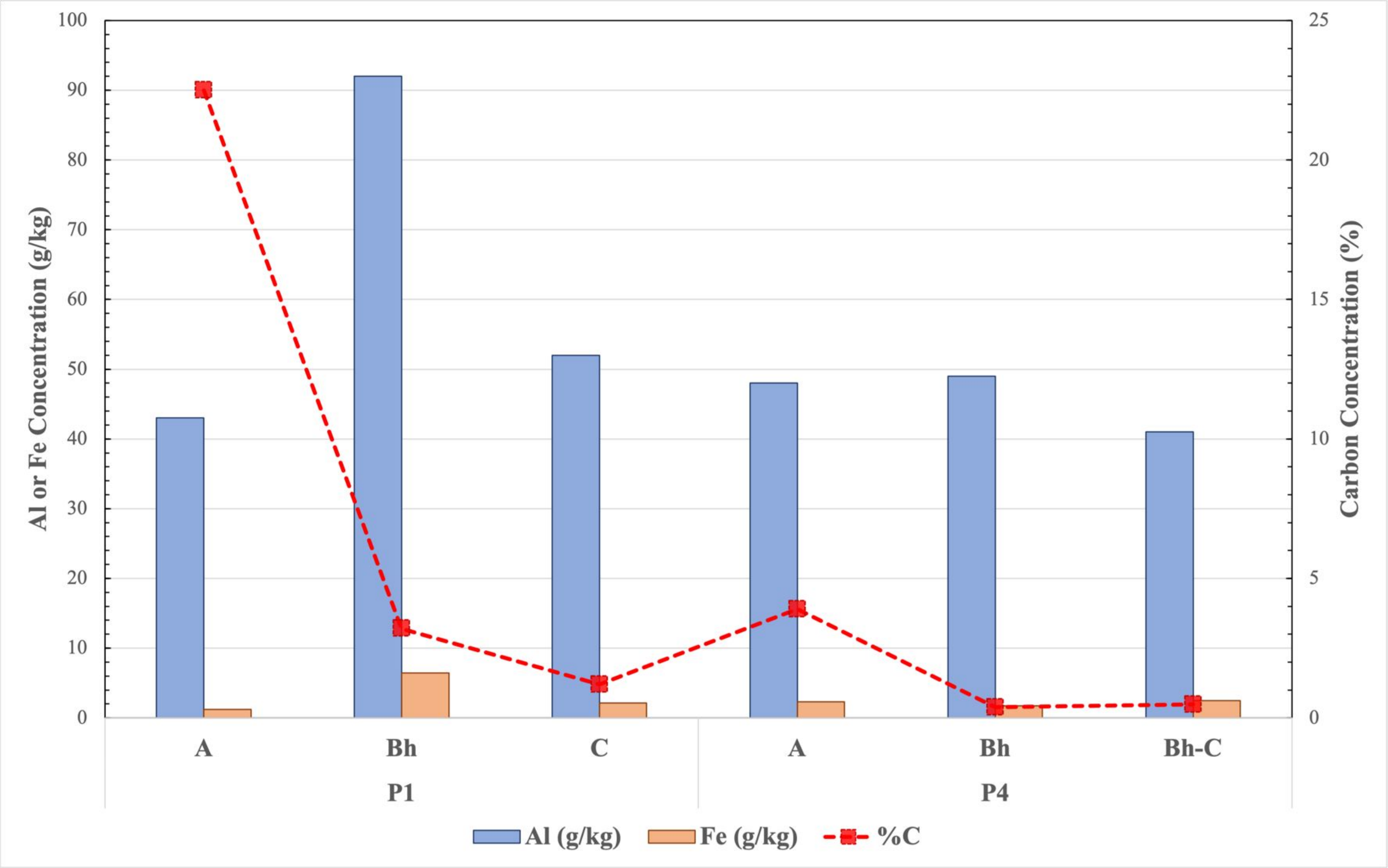

Figure S1. Aluminum (Al, blue bars), iron (Fe, orange bars), and carbon content (%C, red dotted line) in the horizons of the two Amazonian Podzols (P1 and P4).

Elemental composition (C%, H%, and N% contents) was measured in duplicate using ~3 mg of powdered samples on a Perkin Elmer elemental analyzer (model 2400). The calibration standard was acetanilide (C% = 71.09; H% = 6.71 and N% = 10.36). The equipment's detection limits, as determined following the recommendations by Miller and Miller,[2] were C% = 0.04; H% = 0.03; and N% = 0.1. Ash content was determined by heating dried powdered samples at 600 °C for 4 hours in a muffle furnace. The O% content was calculated on an ash-free basis using the following equation: O% = (100 – C% – H% – N % – ash %).

Table S1: Elemental composition of humic and fulvic acids. Additional data for the whole soils can be found in a previous study by Pereira et al. (2013) whereas additional data for humic and fulvic acids can be found in previous studies by Tadini et al.[1] and Tadini et al.[3]

Study area	Horizon	Humic acid (HA) fractions				Fulvic acid (FA) fractions			
		Carbon (%)	Hydrogen (%)	Nitrogen (%)	Oxygen (%)	Carbon (%)	Hydrogen (%)	Nitrogen (%)	Oxygen (%)
**Permanently waterlogged Podzol (P1)**	**A (0 – 15 cm)**	45 ± 3	2.8 ± 0.3	2.3 ± 0.1	50.2	21.4 ±0.5	2.9 ± 0.2	1.2 ± 0.5	74.5
	**Bh (240 cm)**	46 ± 2	3.6 ± 0.2	1.2 ± 0.4	49.2	48.0 ± 0.1	1.4 ± 0.1	1.9 ± 0.4	48.4
	**C (350 cm)**	51 ± 3	4.3 ± 0.1	1.0 ± 0.3	44.0	39.0 ± 0.9	3.1 ± 0.1	2.1 ± 0.2	56.2
**Well-drained Podzol (P4)**	**A (0 – 20 cm)**	45.2 ± 0.3	4.3 ± 0.2	2.9 ± 0.2	47.6	34.4 ± 0.3	7.4 ± 0.5	2.0 ± 0.1	56.2
	**Bh (170 – 180 cm)**	52 ± 6	3.1 ± 0.2	2.6 ± 0.3	42.1	16.1 ± 0.1	3.1 ± 0.1	1.2 ± 0.2	79.6
	**Bh-C (370 – 380 cm)**	53.0 ± 0.3	3.4 ± 0.2	2.4 ± 0.1	41.4	33.0 ± 0.2	6.3 ± 0.3	2.4 ± 0.2	58.5

Section 2. Importance of humic substances in podzolization processes

Humic substances, namely humic acid (HA) and fulvic acid (FA), are central to the formation and evolution of Amazonian podzol soils. Their distinct chemical and physical properties govern metal mobility and complexation, especially for aluminum (Al) and iron (Fe), thus HA and FA play a fundamental role in the vertical transport and stabilization of Al and Fe during podzolization. The properties and importance of HA and FA in Amazonian Podzols are summarized in Table S2 below based on the findings from Tadini et al.[1,3,4]

Table S2: Key differences between Humic and Fulvic acids and their roles in Podzolization (Tadini et al.[1,3,4]; Montes et al.[5]).

	**Humic Acid (HA)**	**Fulvic Acid (FA)**	**Role in Podzolization**
**Composition**	Complex, stable aromatic structures	Simpler structures, more aliphatic	FA contributes to vertical metal transport
**Solubility**	Soluble only in alkaline media	Soluble in both acidic and alkaline media	FA is easily translocated due to its higher solubility
**Complexation**	Fewer ligand sites; less efficient complexation	Higher density of ligand sites (10x more than HA)	FA mobilizes Al; HA mobilizes Fe
**Stability**	Lower stability; less selective for metals	Higher K; more selective towards Al and Cu	FA forms stable metal complexes, enhancing translocation
**Distribution**	Found in deeper, more humified Bh horizons	Present throughout the soil profile; more variable and mobile	FA moves Al to Bh; HA aids Fe accumulation in Bh
**Mobility**	Low mobility; high stability	High mobility; easily interacts with water	FA facilitates the transport of soluble substances

Section 3. Sample preparation:

A basified solution of HA was treated with 200 µL of cation-exchange resin suspension (Dowex 50WX8, 100 – 200 mesh, Acros Organics) and allowed to equilibrate for 10 minutes at ambient temperature to facilitate cation exchange. Following equilibration, the supernatant was carefully recovered. The resin was then washed with 2000 µL of methanol (MeOH), vortexed briefly to extract any resin-retained organics, and allowed to stand for an additional 10 minutes. Subsequently, 500 µL of the methanol supernatant were mixed with 500 µL of the cation-exchanged aqueous solution to result in a 1:1 MeOH:H_2O (v/v) solution at a final dissolved organic carbon concentration of ~50 mg $C \cdot L^{-1}$ (assuming 100% extraction of the organics retained by the resin). The 1:1 MeOH:H_2O ratio was chosen as it is the most common solvent system for ESI-FT-ICR-MS analysis of DOM allowing for data comparability across studies.[6]

For the FA samples, 5000 µL of ultrapure water (Milli-Q, pH 7) were used to solubilize the powdered samples. Then, the solutions were acidified to pH = 2 using dropwise addition of HCl (Fisher, Certified ACS Plus). Solid-phase extraction (SPE) of the acidified FA solutions was performed using Priority Pollutant (PPL) cartridges (Agilent Technologies, Bond Elut PPL, 100 mg styrene divinyl copolymer) following the protocol described by Goranov et al.[7,8] The final eluates were adjusted to a dissolved organic carbon concentration of ~50 mg $C \cdot L^{-1}$ (assuming an extraction recovery of 50%).

Section 4. Mass Spectrometry of Humic and Fulvic Acids

The samples of this study were analyzed after extensive method development during which the sample preparation approaches described in Section 3 above were found to be most optimal for acquiring the highest quality spectra of each different sample matrix. Further details about the method development have been described previously.[7,8] The (-)ESI-FT-ICR-MS instrument itself is tuned with polyethylene glycol, which allows for setting the parameters of the different instrument components (e.g., voltages of ion optics) to yield spectra of most optimal sensitivity, m/z range, and spectral shape. Then, Suwannee River FA (SRFA) standard from the International Humic Substances Society (https://humic-substances.org/) was analyzed to validate the obtained tune conditions. Briefly, the characteristics of the acquired SRFA spectra are matched to the target SRFA characteristics recommended by the recent ESI-FT-MS interlaboratory comparison study.[6] A complete description of the instrument optimization, tuning, and validation with SRFA have been previously published in the supplemental information of Goranov et al.[7,8]

The samples of this study were analyzed as part of two batches (HA samples in batch 1, FA samples in batch 2). Batch 1 contained many samples and spun over ~24 hours. Batch 2 contained less samples and spun only over ~8 hours. SRFA quality control is analyzed about every 8 hours. Thus, four SRFA spectra were acquired along the analysis of the samples of this study - three spectra along batch 1 (1/3, 2/3, 3/3), and one spectrum along batch 2. The whole spectra and nominal mass distributions (Figure S2) as well as the assigned molecular formulas (Figure S3) showed excellent reproducibility, which is critical when datasets are processed using multivariate statistics such as principal component analysis.

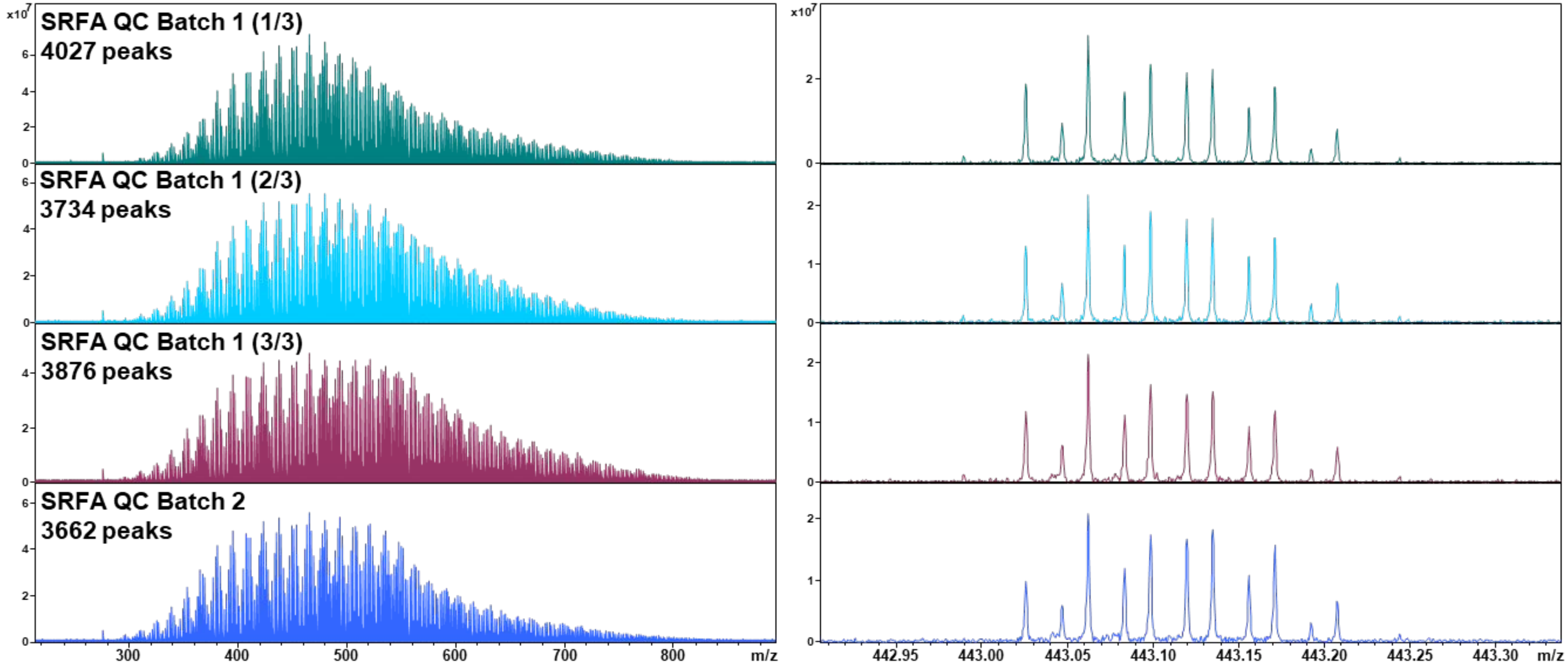

Figure S2. Whole (-)ESI-FT-ICR mass spectra (left) and expanded windows at nominal mass of 443 (right) for Suwannee River fulvic acid (SRFA) quality control (QC) samples showing excellent instrumental reproducibility of the acquired data across the two batches of sample analyses.

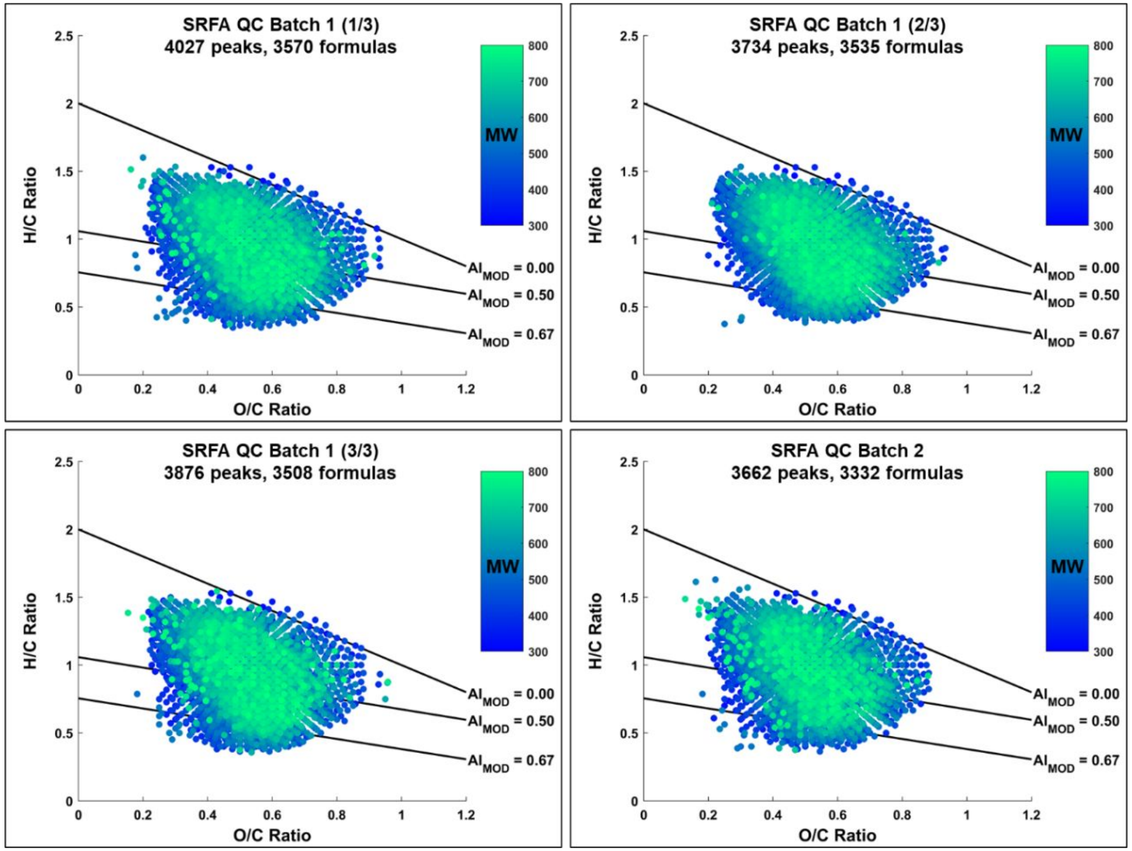

Figure S3. Van Krevelen diagrams (H/C versus O/C molar ratio plots) of assigned molecular formulas to Suwannee River fulvic acid (SRFA) quality control (QC) samples. Formulas are color-coded based on their molecular weight (MW). The black lines indicate modified aromaticity index cutoffs (AI_{MOD}).[9,10]

The SRFA quality control had 3825 ± 162 assignable peaks (i.e., number of peaks obtained after removing blank, salt, and isotopologue peaks),[11] which yielded 3486 ± 106 assigned molecular formulas. The corresponding relative standard deviations are 4% and 3%, respectively, indicating that the data is highly reproducible (below 5%). As the unknown samples of this study were analyzed only once, a 5 % conservative uncertainty was propagated to their data.

Table S3: Number of detected and assigned peaks from (-)ESI-FT-ICR-MS spectra. These values are reproducible within a 5 % uncertainty.

Sample	Horizon	Number of Peaks	Assigned Peaks
**Humic Acids (HA)**			
**Permanently waterlogged Podzol (P1)**	**A (0 – 15 cm)**	3,377	2,661 (79%)
	**Bh (240 cm)**	1,702	1,395 (82%)
	**C (350 cm)**	2,531	2,188 (86%)
**Well-drained Podzol (P4)**	**A (0 – 20 cm)**	3,032	2,521 (83%)
	**Bh (170 – 180 cm)**	3,566	2,495 (70%)
	**Bh-C (370 – 380 cm)**	3,308	2,072 (63%)
**Fulvic Acids (FA)**			
**Permanently waterlogged Podzol (P1)**	**A (0 – 15 cm)**	4,879	3,866 (79%)
	**Bh (240 cm)**	4,110	3,252 (79%
	**C (350 cm)**	4,012	3,221 (80%)
**Well-drained Podzol (P4)**	**A (0 – 20 cm)**	2,627	2,133 (81%)
	**Bh (170 – 180 cm)**	3,046	2,524 (83%)
	**Bh-C (370 – 380 cm)**	3,311	2,661 (79%)

Table S4: Distributions of biochemical classes in Amazonian Podzols. ConAC = Condensed Aromatic Compounds.

Samples	Horizon	Humic Acid (HA)				
		**ConAC (%)**	**Lignin (%)**	**Tannin (%)**	**Aliphatic (%)**	**Others (%)**
**Permanently waterlogged Podzol (P1)**	**A (0 – 15 cm)**	66.10	30.64	1.10	2.02	0.15
	**Bh (240 cm)**	52.27	44.59	1.19	1.96	0.00
	**C (350 cm)**	31.60	61.20	0.50	6.59	0.14
**Well-drained Podzol (P4)**	**A (0 – 20 cm)**	25.98	53.75	0.30	19.38	0.59
	**Bh (170 – 180 cm)**	81.46	9.55	6.61	2.03	0.32
	**Bh-C (370 – 380 cm)**	74.04	13.75	9.09	2.89	0.24
	**Horizon**	**Fulvic Acid (FA)**				
		**ConAC (%)**	**Lignin (%)**	**Tannin (%)**	**Aliphatic (%)**	**Others (%)**
**Permanently waterlogged Podzol (P1)**	**A (0 – 15 cm)**	20.75	61.18	16.22	1.40	0.46
	**Bh (240 cm)**	18.56	60.55	20.62	0.16	0.11
	**C (350 cm)**	16.98	63.80	18.68	0.29	0.24
**Well-drained Podzol (P4)**	**A (0 – 20 cm)**	12.69	69.29	11.92	5.22	0.90
	**Bh (170 – 180 cm)**	14.44	61.93	10.60	11.96	1.07
	**Bh-C (370 – 380 cm)**	23.06	58.59	17.42	0.54	0.37

Section 5. Benzenepolycarboxylic acid measurements of condensed aromatic carbon (ConAC)

Powdered samples were dissolved in basified water at pH 10 (with NH_4OH) with the assistance of sonication. Then, samples were pipetted in 2-mL glass ampules. Additional 0.5 mL of ultrapure methanol were added to ensure no HA/FA solution remained on the walls of the vials. Vials were heated at 60 ^oC until all liquid evaporated leaving known amounts of powdered HA/FA ready for acid thermochemolysis. Then, 2 mL 65 % HNO3 (J.T. Baker, trace metal grade) were added and the ampules were flame-sealed. Thermochemolytic acid digestion was achieved in a programmable oven for 6 hours at 160 ^oC (Ding et al., 2013). Then, HNO_3 was evaporated at 60 ^oC under a stream of ultrapure N_2 gas (Airgas, UHP300). The residue was then dissolved in 1 mL of 0.6 M phosphoric acid and filtered using a 0.2 µm PTFE filter. Benzenehexacarboxylic acid (B6CA) and benzenepentacarboxylic acid (B5CA) were quantified using high performance liquid chromatography with spectrophotometric detection at 254 nm on an Agilent 1100 system. Separation was performed using organic-free eluents of 0.6 M phosphoric acid (pH = 1) and phosphate buffer (20 mM, pH = 6) on an Agilent Poroshell 120 Phenyl-Hexyl (4.6 x 150 mm, 2.7 µm) column, with conditions described previously by Wagner et al.[12] The concentrations of B6CA and B5CA were used to estimate the ConAC content using the scaling factor of 7.04 proposed by Bostick et al.[13]

Table S5: Benzenepolycarboxylic acid measurements of condensed aromatic carbon (ConAC) relative to the organic carbon (OC). B6CA: Benzenehexacarboxylic acid; B5CA: benzenepentacarboxylic acid.

		**Humic Acid (HA)**	
**Samples**	**Horizon**	**ConAC/OC (%)**	**B6CA:B5CA ratio**
**Permanently waterlogged Podzol (P1)**	**A (0 – 15 cm)**	20.61 ± 0.17	0.328 ± 0.008
	**Bh (240 cm)**	21.08 ± 0.32	0.282 ± 0.009
	**C (350 cm)**	17.00 ± 0.27	0.290 ± 0.001
**Well-drained Podzol (P4)**	**A (0 – 20 cm)**	2.97 ± 0.08	0.479 ± 0.006
	**Bh (170 – 180 cm)**	5.78 ± 0.20	0.600 ± 0.029
	**Bh-C (370 – 380 cm)**	2.56 ± 0.00	0.863 ± 0.025
		**Fulvic Acid (FA)**	
	**Horizon**	**ConAC/OC (%)**	**B6CA:B5CA ratio**
**Permanently waterlogged Podzol (P1)**	**A (0 – 15 cm)**	7.18 ± 0.32	0.305 ± 0.012
	**Bh (240 cm)**	36.93 ± 0.00	0.267 ± 0.004
	**C (350 cm)**	33.71 ± 0.54	0.215 ± 0.005
**Well-drained Podzol (P4)**	**A (0 – 20 cm)**	0.63 ± 0.00	0.243 ± 0.002
	**Bh (170 – 180 cm)**	1.68 ± 0.02	0.407 ± 0.006
	**Bh-C (370 – 380 cm)**	1.01 ± 0.04	0.253 ± 0.001

Section 6. Statistical analysis

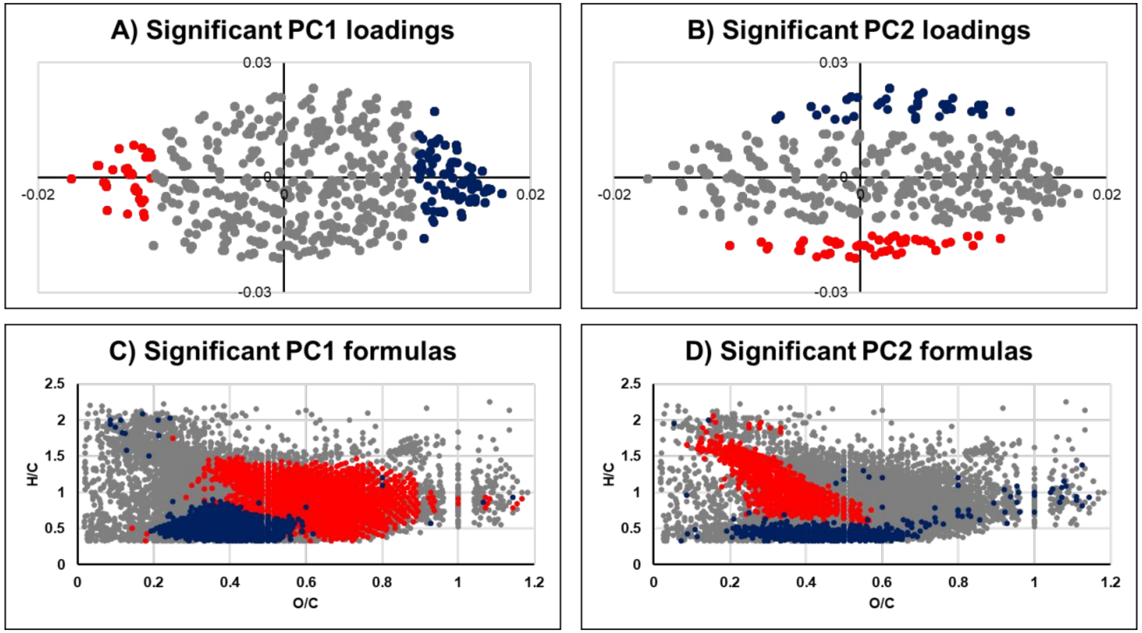

Figure S4. Loadings for principal component analysis (A, B) explored through p-values instead of co-location (as done in the manuscript, Figure 3). The loadings of principal component 1 (PC1) on panel (a) show significant formulas ($p < 0.05$) of fulvic acid samples on the negative end (colored in red) and significant formulas ($p <0.05$) of humic acids on the positive end (colored in blue). The corresponding van Krevelen diagram (C) shows the corresponding formulas agreeing with whole-sample van Krevelen diagrams (Figure 2). Panels B and D show the significant formulas along principal component 2 (PC2). As these formulas are found in both humic and fulvic acid samples, their interpretation is not possible, which deemed the p-value exploration approach as inappropriate and necessitated the use of co-location (as shown in Figure 3). Please note that there are many formulas sharing the same loading values and therefore, the points on panels A and B are of many stacked markers. Thus, it appears that there are fewer loading markers (panels A, B) than formula markers (panels C, D).

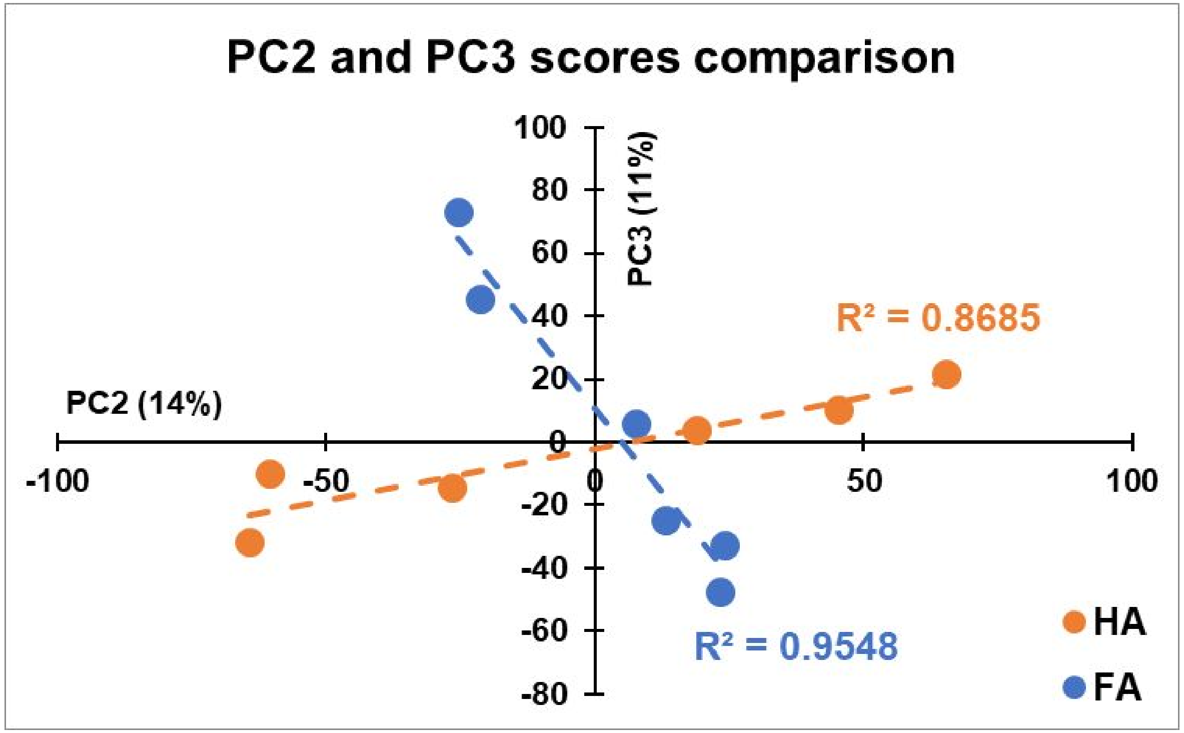

Figure S5. Comparison of sample scores for the second and third principal components (PC2 and PC3, respectively) of humic and fulvic acids revealing no additional insights from PC3, as it is strongly correlated with PC2. Therefore, PC3 results were not used in this study.

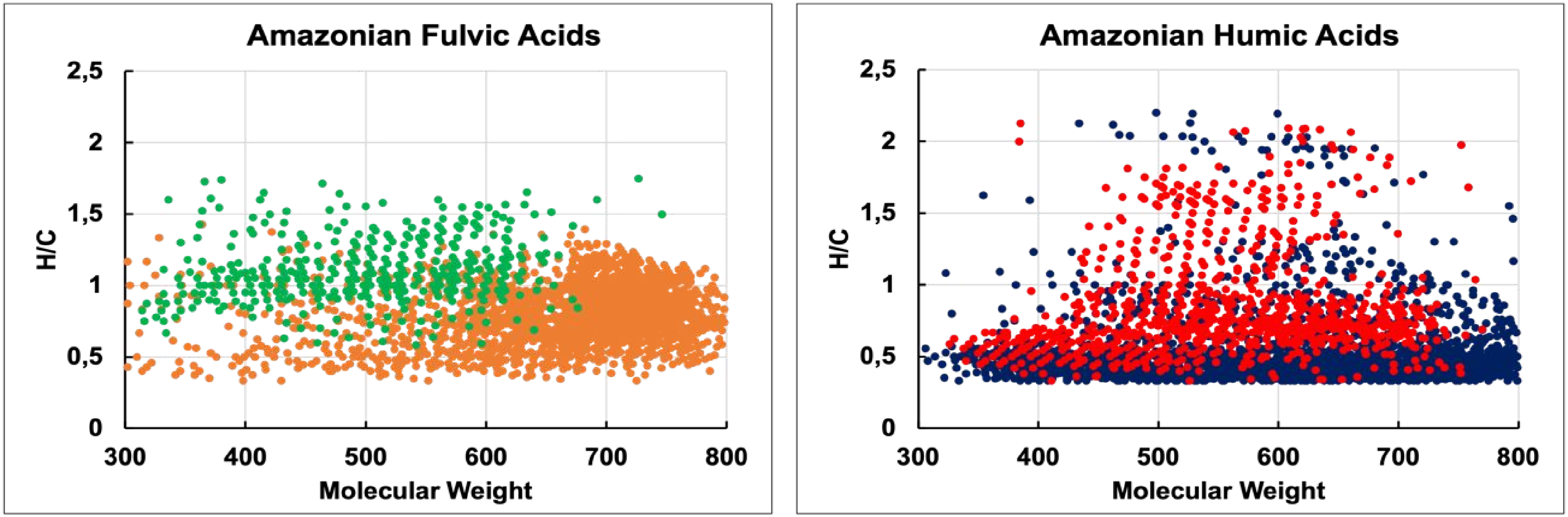

Figure S6. H/C vs. Molecular Weight plots of the significant molecular formulas of fulvic acids (a) and humic acids (b) determined as significant by co-location from principal component analysis. Formulas are colored according to their quadrant location (Figure 3).

References:

1. Tadini, A.M.; Nicolodelli, G.; Marangoni, B.S.; Mounier, S.; Montes, C.R.; Milori, D.M.B.P. Evaluation of the roles of metals and humic fractions in the podzolization of soils from the Amazon region using two analytical spectroscopy techniques. *Microchem J* **2019**, 144, 454-460.
2. Miller, J. C., Miller, J.N. *Statistics for Analytical Chemistry*. Editora Ellis Honwood PTR Prentice Hall, 1993.
3. Tadini, A.M.; Nicolodelli, G.; Senesi, G.S.; Ishida, D.A.; Montes, C.R.; Lucas, Y.; Mounier, S.; Guimarães, F.E.G.; Milori, D.M.B.P. Soil organic matter in podzol horizons of the Amazon region: Humification, recalcitrance, and dating. *Sci Total Environ* **2018**, 613-614, 160-167.
4. Tadini, A.M.; Mounier, S.; Milori, Milori, D.M.B.P. Modeling the quenching of fluorescence from organic matter in Amazonian soils. *Sci Total Environ* **2020**, 698, 1-9.
5. Montes, C.R., Merdy, P., Silva, W.T., Ishida, D., Melfi, A.J., Santin, R.C., Lucas, Y. Mineralization of soil organic matter from equatorial giant podzols submitted to drier pedoclimate: A drainage topochronosequence study. *Catena* **2023**, 222, 1-10.
6. Hawkes, J.A., D'Andrilli, J., Agar, J.N., Barrow, M.P., Berg, S.M., Catalán, N., Chen, H., Chu, R.K., Cole, R.B., Dittmar, T., Gavard, R., Gleixner, G., Hatcher, P.G., He, C., Hess, N.J., Hutchins, R.H.S., Ijaz, A., Jones, H.E., Kew, W., Khaksari, M., Palacio Lozano, D.C., Lv, J., Mazzoleni, L.R., Noriega-Ortega, B.E., Osterholz, H., Radoman, N., Remucal, C.K., Schmitt, N.D., Schum, S.K., Shi, Q., Simon, C.,

Singer, G., Sleighter, R.L., Stubbins, A., Thomas, M.J., Tolic, N., Zhang, S., Zito, P. and Podgorski, D.C. An international laboratory comparison of dissolved organic matter composition by high resolution mass spectrometry: Are we getting the same answer? *Limnol Oceanogr Methods* **2020**, 18, 235-258.

7. Goranov, A.; Tadini, A. M.; Martin-Neto, L.; Bernardi, A. C. C.; Oliveira, P. P. A.; Pezzopane, J. R. M.; Milori, D. M. B. P.; Mounier, S.; Hatcher, P. G. DATASET: Comparison of sample preparation techniques for the (-)ESI-FT-ICR-MS analysis of humic and fulvic acids. *Mendeley Data*, 2022a.
8. Goranov, A.; Tadini, A. M.; Martin-Neto, L.; Bernardi, A. C. C.; Oliveira, P. P. A.; Pezzopane, J. R. M.; Milori, D. M. B. P.; Mounier, S.; Hatcher, P. G. Comparison of sample preparation techniques for the (-)ESI-FT-ICR-MS analysis of humic and fulvic acids. *Environ Sci Technol* **2022b**, 56, 17, 12688-12701.
9. Koch, B.P., Dittmar, T. From mass to structure: An aromaticity index for high-resolution mass data of natural organic matter. *Rapid Commun Mass Spectrom* **2016**, 30(1), 250.
10. Koch, B.P., Dittmar, T. From mass to structure: An aromaticity index for high-resolution mass data of natural organic matter (Erratum). *Rapid Commun Mass Spectrom* **2016**, 30(1), 250.
11. Goranov, A.I., Sleighter, R.L., Yordanov, D.A., Hatcher, P.G. TEnvR: MATLAB-based toolbox for environmental research. *Anal Methods* **2023**, 15, 5390-5400.

12. Wagner, S., Brandes, J., Goranov, A. I., Drake, T. W., Spencer, R. G. M., & Stubbins, A. Online quantification and compound-specific stable isotopic analysis of black carbon in environmental matrices via liquid chromatography-isotope ratio mass spectrometry. *Limnol Oceanogr Methods* **2017**, 15, 12, 995-1006.
13. Bostick, K. W., Zimmerman, A. R., Wozniak, A. S., Mitra, S., & Hatcher, P. G. Production and composition of pyrogenic dissolved organic matter from a logical series of laboratory-generated chars. *Front Earth Sci* **2018**, 6(43), 1-14.
